# Regulation of 3β-Hydroxysteroid Dehydrogenase/Δ^5^-Δ^4^ Isomerase: A Review

**DOI:** 10.3390/ijms140917926

**Published:** 2013-09-02

**Authors:** Martin Krøyer Rasmussen, Bo Ekstrand, Galia Zamaratskaia

**Affiliations:** 1 Department of Food Science, Aarhus University, DK-8830 Tjele, Denmark; E-Mail: martink.rasmussen@agrsci.dk; 2 Department of Food Science, BioCenter, Swedish University of Agricultural Sciences, S-750 07 Uppsala, Sweden; E-Mail: galia.zamaratskaia@slu.se

**Keywords:** hydroxysteroid dehydrogenase, pig, boar taint, expression, steroid hormones, metabolism

## Abstract

This review focuses on the expression and regulation of 3β-hydroxysteroid dehydrogenase/Δ^5^-Δ^4^ isomerase (3β-HSD), with emphasis on the porcine version. 3β-HSD is often associated with steroidogenesis, but its function in the metabolism of both steroids and xenobiotics is more obscure. Based on currently available literature covering humans, rodents and pigs, this review provides an overview of the present knowledge concerning the regulatory mechanisms for 3β-HSD at all omic levels. The HSD isoenzymes are essential in steroid hormone metabolism, both in the synthesis and degradation of steroids. They display tissue-specific expression and factors influencing their activity, which therefore indicates their tissue-specific responses. 3β-HSD is involved in the synthesis of a number of natural steroid hormones, including progesterone and testosterone, and the hepatic degradation of the pheromone androstenone. In general, a number of signaling and regulatory pathways have been demonstrated to influence 3β-HSD transcription and activity, e.g., JAK-STAT, LH/hCG, ERα, AR, SF-1 and PPARα. The expression and enzymic activity of 3β-HSD are also influenced by external factors, such as dietary composition. Much of the research conducted on porcine 3β-HSD is motivated by its importance for the occurrence of the boar taint phenomenon that results from high concentrations of steroids such as androstenone. This topic is also examined in this review.

## 1. Introduction

The elimination of xenobiotics is usually conducted in two phases: Phase I and Phase II. The majority of xenobiotics are Phase I metabolized in the liver by the cytochrome P450 (CYP) enzyme family. However, though receiving less attention, other enzymes are also able to perform Phase I metabolism. One example of such an enzyme is 3β-hydroxysteroid dehydrogenase (3β-HSD), mostly known for its role in steroidogenesis and steroid metabolism. In addition, the porcine version of 3β-HSD has received some attention because of its importance for hepatic clearance of the steroid androstenone in sexually mature male pigs. The accumulation of steroids, especially androstenone, is associated with a negative perception of the meat from pigs.

The enzyme 3β-HSD is a key enzyme in the biosynthesis of all active steroid hormones. Several different isoforms have been identified in several species. Some of these isoforms are expressed in the liver and kidneys or exclusively in the liver [1]. The expression of 3β-HSD in different organs is dependent upon developmental stages [2]. In the liver, 3β-HSD functions in steroid degradation and the regulation of steroid hormone levels in plasma, as a steroid reductase, and also in the Phase I degradation of the male pheromone androstenone [3]. However, its function is not restricted to the inactivation of intrinsic steroid hormones [1]. The activity of 3β-HSD is affected by external factors, such as diet and environmental toxins. Isoflavones, such as genistein and daidzein, and their metabolites have been demonstrated to affect the enzyme kinetics of 3β-HSD in some species [4].

There are also reports detailing the direct metabolic activity of 3β-HSD on xenobiotics, such as the estrogenic mycotoxin zearalenone [5]. Therefore, the regulation of 3β-HSD expression and activity, especially in the liver, is an important aspect of the environmental toxicology of xenobiotics. The body of humans and animals are challenged with zearalenone via the ingestion of foods such as cereals and corn. This compound at high concentrations causes hyperestrogenic effects, such as abortion and infertility. For a review of the acute and long-term toxic effects of zearalenone, see Zinedine *et al.* [6]. A portion of the ingested zearalenone has been demonstrated to be converted into metabolites by 3β-hydroxysteroid dehydrogenase in the livers of pigs and rats [5,7,8].

## 2. 3β-Hydroxysteroid Dehydrogenase

The membrane-bound enzyme 3β-hydroxysteroid dehydrogenase was first described by Samuels *et al.* [9] in 1951 and later fully characterised by The *et al.* [10]. It belongs to the family of oxidoreductases, which act on the CH–OH group with NAD^+^ or NADP^+^ as an acceptor. 3β-HSD is located both in the endoplasmic reticulum and mitochondria, where it catalyses 3β-hydroxysteroid dehydrogenation and Δ^5^- to Δ^4^-isomerisation of the Δ^5^-steroid precursors. This was first demonstrated using plasmid transfection to induce the expression of human 3β-HSD isoenzymes [11].

3β-HSD belongs to a large protein family called the short-chain dehydrogenases/reductases, which includes many representatives with diverse functions and a wide-spread occurrence among living organisms [12–14]. This is in contrast to 3α-HSD, which is a member of the aldo-keto reductase family, characterized by a TIM barrel fold [15–17], which is involved in oxidative cholesterol metabolism and regulated via the LXRα-receptor (a “cholesterol sensor”) [18].

3β-HSD is required for the biosynthesis of all classes of steroid hormones, such as glucocorticoids, mineralocorticoids, progesterone, androgens and estrogens [13]. Enzymes of the 3β-HSD family also catalyze the formation and/or degradation of 5α-androstanes and 5α-pregnanes [2]. Thus, 3β-HSD controls critical steroid hormone-related reactions in the adrenal cortex, gonads, placenta, liver, and other peripheral target tissues [19,20].

It is hypothesized that for every sex hormone, a pair of HSDs exists, which will either convert a potent hormone into its cognate inactive metabolite or *vice-versa*. HSDs often catalyse stereoselective reactions at specific positions of the steroid so that for each sex hormone there is an isoform that either will inactivate a hormone or produce an active ligand. This is achieved either by the reduction of a steroid ketone to a steroid alcohol (reductase activity) or by the oxidation of a steroid alcohol to a steroid ketone (oxidase activity) [21].

## 3. Subcellular Localization

3β-HSD has a dual subcellular localization in that it is found both in the endoplasmic reticulum (ER) and in the mitochondria [22,23]. Within these compartments, histochemical techniques have localized 3β-HSD activity to the smooth ER and mitochondrial cristae [24–26]. 3β-HSD’s subcellular localization pattern is unique in that it displays various degrees of ER and mitochondrial distribution. The relevance of the dual localization is unclear, yet it can be hypothesized that smooth ER-localized 3β-HSD would have greater access to cytosolic steroid precursors.

## 4. Transcriptional Regulation of 3β-HSD

Most studies of 3β-HSD transcriptional regulation have been conducted on human HSD genes [13]. In humans, there are two 3β-HSD isozymes encoded by the *HSD3B1* and *HSD3B2* genes. In mice, six 3β-HSD isozymes have been found [27], and in rats, two isoforms have been found [28]. At present, only one isoform of 3β-HSD has been described in pigs [29].

The gene regulation of 3β-HSD is complex and involves several different factors. During development, testicular 3β-HSD expression appears to be separated from luteinizing hormone (LH) regulation [30]. In early studies it was determined that cyclic adenosine monophosphate (cAMP) and protein kinase-C play a role in the regulation mechanism of 3β-HSD in human choriocarcinoma cells [31], potentially linking the mechanism to a number of endocrine and paracrine regulatory mechanisms. In adult rat testes, primary control of 3β-HSD expression occurs through the action of the LH receptor and the induction of the cAMP second messenger system. LH, forskolin, cAMP, and cholera toxin all induce 3β-HSD mRNA in adult rat Leydig cells [32,33].

The activation of the protein kinase-C pathway induces the generation of cAMP but also causes a near-complete inhibition of the stimulatory effects of human chorion gonadotropin (hCG), LH, forskolin, cholera toxin, and cAMP analogues on 3β-HSD mRNA levels in porcine granulosa cells in culture [34]. The expression of 3β-HSD in the adrenals has long been known to be influenced by adrenocorticotropic hormone and angiotensin II [35].

On the receptor level a number of mechanisms have been suggested:

- It has been demonstrated that the nuclear orphan receptor steroidogenic factor 1 (SF-1) controls the expression of human 3β-HSD type II [36]. Moreover, it has been observed that the overexpression of the SF-1 repressor DAX-1 decreases the expression of 3β-HSD [37].
- Another nuclear receptor known to influence 3β-HSD expression is fetoprotein transcription factor, also known as liver receptor homologue-1 (LRH-1, NR5A2) [38], which is found in the ovaries, testes and adipose tissue. Using a gene-reporter assay, a study demonstrated that co-transfection of LRH-1 with a reporter containing the promoter region of the *3β-HSD* gene resulted in greater reporter activity [39].
- GATA4 and 6 have been demonstrated to be potential activators of the human *3β-HSD* promoter [40]. Moreover, it has been demonstrated that co-transfection of GATA with SF-1 and LRH-1 induce greater promoter activation than SF-1 and LRH-1 alone [41].
- In Leydig cells, 3β-HSD transcription has been demonstrated to be regulated via the LH/hCG-receptor and the promoter androgen response element [42] and regulated in the liver via the receptors estrogen receptor α (ERα), androgen receptor (AR), and cyclin D1 expression [43]. Steroids have been demonstrated to be correlated with the expression of 3β-HSD in pig follicles [44], and in rainbow trout ovaries, 3β-HSD I is down-regulated by estradiol-17β *in vivo* [45]. 3β-HSD expression has also been demonstrated to be induced by peroxisome proliferator-activated receptor α (PPARα) activation in human liver cell lines [46].
- It has been demonstrated using knockout mice that the liver X receptor (LXR) represses 3β-HSD expression in very much the same manner that the constitutive androstane receptor is represses the expression of some of the CYPs. Therefore, the binding of LXR agonists can lead to increased 3β-HSD expression [47].
- Havelock *et al.* [48] found that follicle stimulating hormone (FSH) could stimulate the expression of nerve growth factor-induced clone B, which, in turn, up-regulates 3β-HSD type 2 in the human ovary.

Studies have suggested that androgens can inhibit androgen production by the testes and that this repression can occur at the level of 3β-HSD regulation [49]. Rats treated with hCG displayed an increase in 3β-HSD activity, but R1881 (an androgen agonist) decreased hCG induction, whereas cyproterone acetate (an androgen antagonist) increased activity [50]. Similarly, dexamethasone (a glucocorticoid agonist) addition reduced both basal and cAMP stimulatory effects on 3β-HSD mRNA, but not cytochrome P450c17 activity in mouse Leydig cells [51].

The expression of 3β-HSD is altered by several different compounds, both of endogenous and exogenous origin. The presence of steroids and their regulating factors has been demonstrated to change the expression of 3β-HSD. In a study using porcine hepatocytes isolated from intact male pigs weighing 70 and 92 kg, different response patterns to steroid treatment depending on weight were discovered. In low-weight pigs, estrone sulfate produced no effect on 3β-HSD protein expression, whereas a significant induction was present in the hepatocytes from heavy-weight pigs [52]. Similarly, androstenone produced an increase in protein expression in heavy-weight pigs, whereas it had no effect on low-weight pigs. The suggested agonistic effect of steroids on 3β-HSD expression is not consistent with *in vivo* finding. In pigs, both surgical castration and injection with anti-gonadotropin-releasing hormone (immunocastration) have been demonstrated to increase the mRNA and protein expression of hepatic 3β-HSD [53]. Both castration methods lowered the circulating concentration of testosterone and androstenone.

Compounds such as glucocorticoids and interleukins have also been demonstrated to regulate 3β-HSD expression [54–56].

In summary, these findings indicate that the regulation of 3β-HSD is very complex, affected by several compounds and most likely different between tissues, different ages of organisms and species.

## 5. 3β-HSD Isoforms in the Liver

3β-HSD was purified from human liver by Takikawa *et al.* [57,58]. The adult mouse liver expresses 3β-HSD types II, III, and IV, with type III predominating. Mouse type I expression ends at postnatal Day 20, whereas type V is detected at postnatal Day 40 and is male specific, and type II is expressed at low levels throughout development. These data suggest that the mouse liver enzyme might play a key role during foetal development [59]. Rat type III is expressed in the male rat liver and has a function in inactivating steroids in this tissue [60–62].

Studies have also examined the regulation of 3β-HSD in the rodent liver. It appears that isoform-specific, sexually dimorphic regulation of 3β-HSD occurs in rats and mice [61,63].

In the mouse and rat liver, sexually dimorphic GH expression regulates many drug-metabolizing and steroid-metabolizing enzymes [64–68]. GH expression in transgenic mice down-regulates the male-specific isoform of 3β-HSD [69]. These data suggest that circulating levels of steroids might affect the regulation of 3β-HSD activity in the liver, principally through altering GH and PRL levels, thereby resulting in steroid degradation feedback. This periodic variation in GH levels as a wave-formed transmitter-receiver signaling pattern is a general principle for gender differences and should be related to intracellular receiving “oscillators” with specific resonance characters [67,68].

Immunohistochemical studies localized 3β-HSD protein to the bile duct epithelium, and 3β-HSD activity has been mostly associated with the microsomal fraction (containing e.g., ER) [70,71]. Porcine hepatic 3β-HSD cDNA has been sequenced (GenBank accession number: NM_001004049). The gene has been mapped to the chromosome 4q16–4q21 [29]. The structure of the porcine *3β-HSD* gene was described by Cue *et al.* [72]. The protein encoded by the porcine gene displays most similarity to human 3β-HSD type I (373 aa) and murine types I, II, II and IV.

## 6. 3β-HSD in Testicular Leydig Cells

The fluctuations in testicular steroid synthesis might be due to the changes in steroidogenic enzymes. As we have stated above, 3β-HSD is one of the key enzymes in the biosynthesis of androgens and almost all other biologically active steroids. Therefore, high 3β-HSD activity in the testes is essential for normal steroidogenesis and subsequently for reproduction.

The majority of studies on the steroidogenic enzymes have used the Leydig cells of mice and rats as a model, whereas less information is available on the regulation of steroidogenic enzymes in porcine Leydig cells. In the Leydig cells of rats, mRNA for 3β-HSD is expressed at relatively high levels [32]. The expression of 3β-HSD during development is an indicator of testicular androgen production. Adult Leydig cells arise postnatally and encompass three developmental stages: progenitor, immature, and adult cells [73]. Rat testes at postnatal day 15 display 3β-HSD localization to the smooth ER of precursor Leydig cells and endothelial cells near 3β-HSD-positive Leydig cells [74,75]. Pelletier *et al.* [76] have suggested that in the adult rat testes, 3β-HSD is restricted to the mitochondria.

Clark *et al.* [77] studied Leydig cells from immature pigs and demonstrated that mRNA expression for 3β-HSD in porcine Leydig cells is lower than that for the CYP side-chain cleavage enzyme and CYP 17α-hydroxylase/C17-20-lyase. The same *in vitro* study revealed that treatment with hCG resulted in an increase in the mRNA for 3β-HSD in a dose- and time-dependent manner, indicating the regulation of 3β-HSD by gonadotropins as well as by some steroids.

Other localization studies have examined 3β-HSD by immunocytochemistry in cynomolgus monkey testes, showing that 3β-HSD expression is observed in both Leydig and Sertoli cells [78]. Thus, the predominant site of 3β-HSD expression in the testes is in Leydig cells, but evidence exists for a parallel localization in Sertoli cells, at least in some primate species.

The presence of 3β-HSD in pig testicular cytosol was first described by Nakajin *et al.* [79]. The expression of 3β-HSD in pig Leydig cells was localized to the theca interna and was unaffected by follicle atresia [80]. Therefore, the steroidal regulation of 3β-HSD in pig Leydig cells may act through a mechanism separate from the androgen receptor [77]. The expression of 3β-HSD, CYP450c17 and CYP450scc have been analyzed in several porcine fetal tissues, and the expression of 3β-HSD was found to be greater in fetal testes than in the fetal adrenal gland at all stages of development [81].

Studies have been performed to determine whether 3β-HSD regulation in fetal testes might be mediated through growth factors. Epidermal growth factor (EGF), transforming growth factor β (TGF-β), activin A, and GH have been shown to increase 3β-HSD activity. It has also been demonstrated that EGF treatment of porcine Leydig cells increases 3β-HSD activity [82]. Phospholipids may also play a role in the regulation of 3β-HSD activity as demonstrated in *in vitro* microsomal studies [83].

## 7. 3β-HSD in Other Tissues

3β-HSD is expressed in a great number of tissue types in the body: adrenal, ovary, testis, placenta, liver, breast, prostate, skin, brain, kidney, cardiovascular, blood cells, and adipose [13]. Of special interest is the interplay between adrenals and gonads in steroid hormone metabolism [84].

The adrenals undergo a morphological differentiation from the fetal to the adult adrenal cortex. For non-primates, such as rats, guinea pigs and mice, there is no production of androgens in the adrenals due to the lack of cytochrome P450C17 [85]. Therefore, there are no detectable androgens in the serum in these animals when they are castrated. 3β-HSD is, however, expressed in the pig adrenals [81].

For the ovary, differential regulation of 3β-HSD plays an important role in the steroidogenic profile of ovarian tissue, and a complex interplay of pituitary and ovarian factors together maintains a tight control over the enzyme activities involved. Different species have different expression patterns during the development of the various tissues and during the oestrous cycles. The expression of 3β-HSD in pig ovaries was first described by Conley *et al.* [86] (for a review, see [87]). Similar to rats, 3β-HSD is localized to the theca interna layer of the pig follicle [88,89]. The pituitary control of steroidogenesis in the follicles is mainly mediated by FSH and LH, which causes an increase in cAMP and phosphatidylinositol turnover, resulting in an increase in the expression of 3β-HSD and other steroidogenic enzymes [90–92]. Other important factors for 3β-HSD expression in the ovaries are progesterone and androgen, and the connection between the receptors for these steroids and the expression of 3β-HSD has been found at some stages in the *corpus luteum* development [93–95]. Prostaglandin F2α and E2 have been observed to affect the accumulation of progesterone and 3β-HSD mRNA in porcine luteinised granulosa cells [96]. In the *corpus luteum*, the mitochondrial 3β-HSD is especially important for steroidogenesis because of the co-localization with P450scc [97]. mRNA for 3β-HSD and other proteins has been measured in porcine follicles [88]. Increased expression throughout follicular development was observed.

Adipose tissue plays an important role in the metabolism of sex steroids and has been shown to exhibit 3β-HSD activity [98]. One 3β-HSD isoform was characterized in porcine adipose tissue [29], which strongly implies that androstenone is metabolized in adipose tissue. The observed accumulation of androstenone in adipose tissue will then be dependent on the balance of supply and local turn-over.

## 8. Dietary Influence on 3β-HSD

A major gap in the current knowledge of the role of 3β-HSD is the extent to which nutrition interacts with genetics to influence 3β-HSD expression and activity and thus steroid levels. Some dietary components, such as spearmint (*Mentha spicata*), have been demonstrated to have an anti-androgenic effect on male rats, including depressed 3β-HSD activity [99]. Chen *et al.* [100] demonstrated that androstenone levels in fat slightly decreased after the consumption of raw potato starch for 2 weeks. Dietary components most likely accelerated androstenone hepatic metabolism and therefore reduced the concentrations in fat. In another study, chicory root affected both global androstenone levels in male pigs and the levels of 3β-HSD expressed in the liver [101].

Wang *et al.* [102] studied the effects of dietary soybean isoflavone on zearalenone residues in the muscle and liver of young female pigs and demonstrated that addition of zearalenone into the diet increased protein expression of 3α/3β-HSD. Interestingly, the addition of isoflavones to the zearalenone-contaminated diet decreased the hepatic protein expression of 3α/3β-HSD compared with the addition of zearalenone alone. Furthermore, the isoflavones daidzein, genistein, biochanin A and formononetin have been demonstrated to inhibit bovine adrenal 3β-HSD activity [103].

Generally, dietary fats can influence the fatty acid composition of testicular lipids as well as the metabolism of testicular steroids and their concentrations in urine and plasma [104–106]. Testicular 3β-HSD in rats has been demonstrated to be affected by dietary lipid composition [107].

Drugs can also influence 3β-HSD. For example, ciprofibrate fed to male rats dramatically decreases testicular 3β-HSD activity without changing either 3β-HSD protein or mRNA expression [108], which indicates a direct inhibition on enzyme activity level.

## 9. The Boar Taint Phenomenon

The sensory quality of meat originating from entire non-castrated male pigs may be negatively affected by the presence of high concentrations of skatole and/or androstenone. This phenomenon is known as boar taint [109]. The major factor determining the occurrence of boar taint in pig products is the balance between the biosynthesis and catabolism of androstenone and skatole [110]. Skatole is synthesized in the intestine of pigs and catabolized in the liver [110]. Androstenone is synthesized in the Leydig cells of the testes [111,112]. The production of androstenone is, like other sex steroids, mainly regulated by LH excreted by the pituitary gland. A portion of the androstenone is catabolized in the testes, whereas the rest is degraded in the liver. The catabolism of androstenone is conducted in two phases. Phase I is conducted by the enzymes *3α-* and *3β-HSD* with the production of 3α- and 3β-androstenol, respectively [3]. These metabolites undergo Phase II sulpho-conjugation by hydroxysteroid sulphotransferases (isoform 2A1 and 2B1). Both testicular production and liver metabolism are important in the determination of the final concentrations of androstenone in fat. The enzymes responsible for the Phase I metabolism of skatole are to a great extent regulated by the presence of androstenone [113–119]. Cue *et al.* [72] detected a number of polymorphisms in the porcine *3β-HSD* 5′-flanking region. However, none of these has been associated with variations in androstenone levels. This is in contrast to new results indicated that single nucleotide polymorphisms in the full-length gene have an impact on the accumulation of androstenone in Duroc pigs [120].

At present, the regulation of porcine 3β-HSD in the testes is not fully elucidated. In principle, there are hypothetically two ways of connecting 3β-HSD to androstenone levels in male animals: Either inhibiting testis activity and thereby decreasing androstenone synthesis or increasing liver activity and thereby increasing androstenone degradation. There are indications that this second effect can be achieved via bioactive components in the feed, e.g., chicory root [101].

Interactions between 3β-HSD and anabolic steroids in relation to other physiological function in the animals should be established. Recent advances in biochemistry and molecular biology provide us with an excellent opportunity to increase our understanding of 3β-HSD regulations and the methods of its manipulation without side-effects.

## 10. Conclusions

3β-HSD is essential for both steroidogenesis and steroid degradation because of its dual functionality and depending on its localization in Leydig cells or hepatocytes. The molecular regulation of the human and rodent isoenzymes is complex and involves several factors and receptors, whereas the regulatory mechanisms of the porcine 3β-HSD are only starting to be elucidated.

The expression and activity of 3β-HSD depend on the presence of several endogenous compounds, such as steroids, as well as exogenous toxic compounds, such as zearalenone. Moreover, dietary compounds have been demonstrated to affect the expression of 3β-HSD in various organs, including the liver.

3β-HSD is a key enzyme in the metabolism of the boar taint compound androstenone in male pigs. Based on existing data, it is clear that the availability of androstenone depends on the balance between androstenone biosynthesis in the testes and the activities of 3β-HSD in the liver.
